# COVID‐19 and neglected tropical diseases among refugees: Plight of a vulnerable population in Africa

**DOI:** 10.1002/puh2.68

**Published:** 2023-02-15

**Authors:** Isaac Olushola Ogunkola, Molly Unoh Ogbodum, Glory Mbe Egom Nja, Ekpereonne Babatunde Esu

**Affiliations:** ^1^ Global Health Focus Africa Abuja Nigeria; ^2^ Students for Sensible Drug Policy International Vienna Austria; ^3^ Department of Public Health University of Calabar Calabar Nigeria; ^4^ UNESCO International Centre for Biotechnology Nsukka Nigeria

**Keywords:** Africa, COVID‐19, global health, mobile population, NTDs, pandemic, refugee, vulnerable population

## Abstract

Neglected tropical diseases (NTDs) are widespread in tropical and subtropical climates and caused by bacteria, fungi, parasites, and viruses. These infections have devastating physical, social, and economic impacts worldwide, especially in underveloped communities. Migrant populations, such as refugees who may carry diseases or are exposed to them for the first time, face poverty, famine, and bad living conditions. The pandemic's twofold burden and long‐term impact on NTDs among African refugees are highlighted in this article. Sub‐Saharan Africa accounts for roughly 90% of the disease burden due to poverty and some NTDs’ special propensity to thrive in its climates. After a disruption, such as displacement due to war, infectious disease transmission will rise until it reaches pre‐intervention levels if treatments are not started. COVID‐19 threatens NTD control and elimination efforts, which are now directly affected by it. Due to high population density, social isolation is difficult in most African refugee camps. In such circumstances, poor sanitation and lack of access to water make hand hygiene practically impossible. Over the past few decades, refugees and their settlements and camps have faced socioeconomic and health challenges. In order for vulnerable populations, like the refugees, not to be left behind, they should not be forgotten and countries should prepare for the revival of community‐based engagement.

## INTRODUCTION

Neglected tropical diseases (NTDs) have been described as a diverse group of bacterial, parasitic, and viral diseases affecting more than one billion people [1]. These diseases cost developing economies billions of dollars every year with low‐income countries disproportionately affected by ≥5 NTDs simultaneously [1, 2]. The World Health Organization (WHO) increased the list of prioritized diseases to 20 in 2017 [1]. These diseases include dracunculiasis, human African trypanosomiasis, visceral filariasis, trachoma, soil‐transmitted helminthes, leishmaniasis, leprosy, onchocerciasis, schistosomiasis, Buruli ulcer, rabies, scabies, and snakebite envenomation among others [1].

Throughout the world, NTDs cause devastating health, social, and economic consequences, and impoverished communities are heavily burdened [1]. Although this group of diseases lingers, the emergence of coronavirus disease (COVID‐19) that is zoonotic in nature has just increased the stakes exponentially. It is inevitable that the economic crisis will also drag millions of people into poverty envisaging its effect on the large populations affected and at risk to NTDs [2]. The toll of NTDs on global health is far‐fetched as they have a great impact on the poor in tropical regions. NTDs are influenced by water, sanitation, and hygiene (WASH) conditions that necessitate joint implementation, management, and evaluation of NTDs and WASH programs [1].

Undebatably, conditions of poverty and malnutrition are prevalent, and living conditions are grim among migrating populations such as refugees who are exposed to infections for the first time or who introduced diseases into new areas. Furthermore, the chaos and instability often lead to poor living conditions, which further exacerbate the risk to rapid transmission of infectious diseases [2]. This commentary highlights the dual burden of the pandemic alongside its compounding and long‐lasting effect of NTDs among refugees in Africa.

## IMPACT OF COVID‐19 ON NTDS IN AFRICA

Humanity faces a series of public and global health issues. Vulnerable populations throughout the world as well as marginalized communities in low‐ and middle‐income countries not only have less access to health care but are also disproportionately affected by a wide range of NTDs. About 90% of the disease burden can be linked to sub‐Saharan Africa. This is owing to widespread poverty and the discrete features of some NTDs to thrive in specific climates [3]. During a disruption to interventions, the transmission of infectious diseases will gradually rise back toward the pre‐intervention level unless interventions are reintroduced. The resulting rate of resurgence in prevalence depends on the rate at which new infections occur, which is dependent on the natural history of the disease and the local transmission conditions. Resurgence rates for many NTDs are relatively slow, particularly when compared with other infectious diseases, meaning that the impact of a short disruption to NTDs programs will accrue gradually [4].

COVID‐19 threatens the hard‐won gains in NTDs control and elimination efforts and has become a conduit through which NTDs programs are now being affected instantaneously. NTDs interventions have been revealed to be among the most often and profoundly impacted by the COVID‐19 pandemic throughout the whole range of vital health services [5]. This resultant effect is evident in the disruptions of most NTDs‐based community programs. Chiefly, the suspension of mass treatment/preventive chemotherapy as Ghana employed a large number of community health workers in COVID‐19 contact tracing led to a reduction in human resources and delays in diagnosis, treatment, morbidity management, and disability prevention of NTDs [4]. The modeling of WASH on NTDs transmission has received limited attention. COVID‐19 restrictions and lockdown measures impeded WASH awareness programs as well as health education campaigns, active case finding, and vector control measures. Vector control offers the potential to mitigate the impact of a disruption to programs as it reduces the transmission rate of some infections, therefore slowing resurgence. Although this is no longer feasible, efforts should be directed toward the resumption of vector control activities [4, 5]. Services, such as rehabilitation, support for self‐care, and psychosocial services, are equally unobtainable [5].

As resources are constrained, the COVID‐19 pandemic has strained the finances of governments, nongovernmental organizations, and humanitarian agencies [4]. Financial resources considered necessary for NTDs interventions are now being diverted to target the pandemic. This has left little or no cognizance attached to the burden of NTDs in resource‐limited settings as is the case in most countries in Africa. Human resources are as well deflected as NTDs personnel are reassigned to support COVID‐19 response, and the availability of care workers to manage comorbidities is limited as a result of absence from work due to illness or government‐authorized movement restriction [5].

A myriad of uncertainties exist ahead with the pandemic in play, and improved health outcomes obtainable per time are hindered by these underlying factors influenced by the pandemic that aggravates health issues. Noticeable delay has been observed in the manufacture, conveyance, and delivery of NTDs medicines and consumables within African countries [5]. Likewise, diagnosis, treatment, and care of NTDs affected populations, and other health facility‐based services are now being hampered [5]. Prioritization and rationalization of mitigation methods can be utilized to react to programmatic demands, improving NTDs programs for the future, in the context of limited resources for NTDs programs and the multifaceted impact of COVID‐19 on health systems [4,6].

## THE PLIGHT OF VULNERABLE AFRICAN REFUGEES

According to the Office of the United Nations High Commissioner for Refugees (UNHCR), the world is presently being faced with the utmost levels of displacement ever in history [6, 7]. With an unmatched 65.3 million people forced to leave their homes as a result of war, conflicts, drought or poor economies, and the vulnerable population have variable access to health care in the country in which they reside [7]. Africa contributes a substantial number of refugees and migrants due to natural disasters, economic crises, and wars on the continent. From 2000 to 2017, it is important to note that Africa accounted for about 14.7 million international migrants making it the second continent after Asia, which had the highest number of international migrants in the year in review [7]. There were a total of 118,374,355 refugees from different parts of Africa between the years 1990 and 2017 [7]. The yearly increase in the numbers of refugees in Africa and some other parts of the world can be associated with some factors. These factors or drivers of refugee migration in Africa include natural disasters, climate change, violence and crises, and economic hardship [7]. Violent conflict and crises, such as the Boko Haram terrorism in Nigeria, the Anglophone–Francophone conflicts in Cameroon, and the Fulani attacks in Niger, are some of the predominant drivers of refugees in those regions [7].

The importance of WASH services cannot be undermined in infection and prevention control (IPC) [8]. Many refugees in Africa face challenges accessing basic services, including WASH services, which consequentially escalate their risk of morbidity and mortality [8]. The prevalence of disease burden highlights high levels of poor health and associated social burden among refugee populations, which are very much connected to insufficient WASH facilities and lack of access to clean water [6]. The prevalence of NTDs and/or diarrheal disease in refugee camps is often significantly higher than in their host countries [9]. Evidence has substantiated that handwashing with soap and water can reduce the risk of diarrheal disease by 23%–40% [9]. Equally, inconsistent soap availability for handwashing has been shown to be conventional in most African refugee camps and settlements [6].

Most NTDs are generally preventable, even without vaccines. WASH facilities, good hygiene, and sanitary food handling can prevent diseases, such as soil‐transmitted helminths, schistosomiasis, and trachoma. Moreover, appropriate handwashing and sanitizing have proven to be useful in the IPC of COVID‐19. But the availability of these preventable facilities and uptake of the needed behavior is suboptimal in most refugee settlements [9]. NTDs are intimately concomitant to poverty, in areas where access to adequate sanitation, clean water, and medical care is limited [8]. It is no coincidence that the majority of the burden of these diseases is in neglected and impoverished communities such as the refugee settlements in Africa [10]. With NTDs endemic in most refugee settlements, the advents of COVID‐19 ushered a distraction in health responses globally, making NTDs forgotten diseases of forgotten people—refugees. Millions of refugees in Africa are exposed to family separation, violence, and exile [6]. The COVID‐19 pandemic exposes these populations to a new threat, one that could prove to be more overwhelming than the initial events compelling them to abscond from their countries [9]. Although efforts toward curbing the widespread of NTDs are underway in other populations, there has been no such effort targeted at refugees amidst the pandemic yet they remain pliable to infections; this defies the mantra of the sustainable development goals of “leaving no one behind” and failure to control or eliminate NTDs will be a direct reflection of our inability to respond to the needs of the most disadvantaged and neglected populations [1].

Table 1 shows that there are over one million refugees living in the 13 priority African countries listed by WHO for COVID‐19. As described in the table, some of these countries have an increased number of COVID‐19 cases and at the same time are burdened with 4–5 NTDs simultaneously, such as Ethiopia. Refugees are no doubt vulnerable to COVID‐19, as they reside in settings that extremely increase their risk of contagion. For instance, most refugee settlements in Africa are densely populated, and social distancing in such conditions is challenging besides if basic sanitation is lacking, proper hand hygiene is near to impossible in such settings [6, 9, 10]. Similarly, the preventive measures employed during the COVID‐19 era are in tandem with NTDs preventive measures. The impact of COVID‐19 did not just worsen the state of affairs in the refugee settlements but also diverted the needed health resources for endemic NTDs in those settings toward combating the pandemic [9, 10]. This development hampered the progress made so far toward combating NTDs as the new road map for NTDs for the period 2021–2030 was intended to be officially launched in June 2020 but was postponed to January 2021 due to the COVID‐19 pandemic making the vulnerable population—refugees in Africa faced with a double plight [1].

**TABLE 1:** Population distribution of the World Health Organization (WHO) identified 13 priority African countries for COVID‐19, total cases, total number of refugees, and endemic neglected tropical diseases (NTDs) in the countries.

Countries	Population as at mid‐2020 in million	Total number of reported COVID‐19 cases (April 2022)	Total number of refugees in the country as at 2020	Endemic NTDs in the countries
**Algeria**	44.4	265,727	4693	Soil‐transmitted helminthes (STH) and trachoma
**Angola**	32.5	99,194	8264	Lymphatic filariasis (LF), onchocerciasis, schistosomiasis, STH, and trachoma
**The Democratic Republic of Congo**	89.6	86,747	840,449	LF, onchocerciasis, schistosomiasis, and STH
**Cote d'Ivoire**	26.2	81,829	37,601	LF, onchocerciasis, STH, schistosomiasis, and trachoma
**Ethiopia**	114.9	470,096	151,336	LF, onchocerciasis, STH, schistosomiasis, and trachoma
**Ghana**	31.1	161,071	13,887	LF, onchocerciasis, trachoma, schistosomiasis, STH, Buruli ulcer, yaws, leprosy, guinea worm, human African trypanosomiasis (HAT), cutaneous leishmaniasis (CL), and rabies
**Nigeria**	206.1	255,663	352,953	LF, onchocerciasis, STH, schistosomiasis, trachoma, leprosy, Buruli ulcer, and HAT
**Mauritius**	1.3	214,860	191	STH
**South Africa**	59.6	3732,628	494	STH, schistosomiasis, leprosy, and rabies
**Tanzania**	59.7	33,851	714	LF, onchocerciasis, STH, schistosomiasis, leprosy, and rabies
**Kenya**	53.5	323,557	7452	LF, STH, schistosomiasis, and trachoma
**Uganda**	45.7	163,932	7390	Visceral leishmaniasis (VC), HAT, trachoma, Buruli ulcer, STHs, schistosomiasis, LF, and onchocerciasis
**Zambia**	18.4	317,804	269	LF, STH, schistosomiasis, and trachoma
**Total**	783	6206,959	1425,693	

## RECOMMENDATIONS AND CONCLUSION

There has been a rise in the population of refugees over the last decades with increasing socioeconomic and health‐related problems in their settlements and camps. One of the vulnerable populations paying the highest price for COVID‐19 is the refugees who are disproportionately faced with inherent health challenges such as NTDs. Surprisingly, most NTDs and COVID‐19 can be prevented with proper social distancing or by avoiding overcrowding, and the use of well‐equipped WASH facilities. The population has struggled to beat NTDs, and the dawn of COVID‐19 exacerbates hard‐won gains made toward the eradication of these forgotten diseases.

Countries should gear up toward the resumption of community‐based intervention, especially for the vulnerable population such as the refugees. COVID‐19 information and vital statistics should be integrated into the existing NTDs platforms to build a robust database for evidence‐based interventions. There should be a synergized agenda to beat both COVID‐19 and NTDs reaching all those in need with the prevention, treatment, and care, including refugees. Moreover, there is a paucity of research on NTDs among refugees. Research institutions should be encouraged to explore tropical diseases among this population in comparison to their host countries.

## AUTHOR CONTRIBUTIONS

Isaac Olushola Ogunkola and Molly Unoh Ogbodum conceived the idea, wrote the draft of the manuscript, and collected data and literature. Ekpereonne Babatunde Esu and Glory Mbe Egom Nja assisted with data collection, article interpretation, and language edit. The authors read and approved the final manuscripts.

## CONFLICT OF INTEREST STATEMENT

Isaac Olushola Ogunkola is an Editorial Board member of Public Health Challenges and a coauthor of this article. He was excluded from editorial decision‐making related to the acceptance of this article for publication in the journal.

## CONSENT FOR PUBLICATION

The authors all agreed to the publication of this manuscript.

## FUNDING INFORMATION

Not applicable.

## ETHICS STATEMENT

Not applicable.

## Data Availability

Not applicable.
